# A deep adversarial variational autoencoder model for dimensionality reduction in single-cell RNA sequencing analysis

**DOI:** 10.1186/s12859-020-3401-5

**Published:** 2020-02-21

**Authors:** Eugene Lin, Sudipto Mukherjee, Sreeram Kannan

**Affiliations:** 1 Department of Electrical & Computer Engineering, University of Washington, Seattle, WA 98195 USA; 2 Department of Biostatistics, University of Washington, Seattle, WA 98195 USA; 3 Graduate Institute of Biomedical Sciences, China Medical University, Taichung, Taiwan

**Keywords:** Adversarial autoencoder, Variational autoencoder, Dimensionality reduction, Generative adversarial networks, Single-cell RNA sequencing

## Abstract

**Background:**

Single-cell RNA sequencing (scRNA-seq) is an emerging technology that can assess the function of an individual cell and cell-to-cell variability at the single cell level in an unbiased manner. Dimensionality reduction is an essential first step in downstream analysis of the scRNA-seq data. However, the scRNA-seq data are challenging for traditional methods due to their high dimensional measurements as well as an abundance of dropout events (that is, zero expression measurements).

**Results:**

To overcome these difficulties, we propose DR-A (Dimensionality Reduction with Adversarial variational autoencoder), a data-driven approach to fulfill the task of dimensionality reduction. DR-A leverages a novel adversarial variational autoencoder-based framework, a variant of generative adversarial networks. DR-A is well-suited for unsupervised learning tasks for the scRNA-seq data, where labels for cell types are costly and often impossible to acquire. Compared with existing methods, DR-A is able to provide a more accurate low dimensional representation of the scRNA-seq data. We illustrate this by utilizing DR-A for clustering of scRNA-seq data.

**Conclusions:**

Our results indicate that DR-A significantly enhances clustering performance over state-of-the-art methods.

## Background

Dimensionality reduction is a universal preliminary step prior to downstream analysis of scRNA-seq data such as clustering and cell type identification [1]. Dimension reduction is crucial for analysis of scRNA-seq data because the high dimensional scRNA-seq measurements for a large number of genes and cells may contain high level of technical and biological noise [2]. Its objective is to project data points from the high dimensional gene expression measurements to a low dimensional latent space so that the data become more tractable and noise can be reduced. In particular, a special characteristic of scRNA-seq data is that it contains an abundance of zero expression measurements that could be either due to biological or technical causes. This phenomenon of zero measurements due to technical reasons is often referred to as “dropout” events where an expressed RNA molecule is not detected. The identification of distinct cellular states or subtypes is a key application of scRNA-seq data. However, some methods may not work well because of the existence of dropout events.

The most commonly used method is principal component analysis (PCA), which transforms the observations onto the latent space by defining linear combinations of the original data points with successively largest variance (that is, principal components) [3]. However, PCA is under the assumptions of linear dimensions and approximately normally distributed data, which may not be suitable for scRNA-seq data [4]. Another linear technique is factor analysis, which is similar to PCA but aims to model correlations instead of covariances by describing variability among correlated variables [5]. Based on the factor analysis framework, a recent state-of-the-art method, Zero-Inflated Factor Analysis (ZIFA), accounts for the presence of dropouts by adding a zero-inflation modulation layer [6]. A limitation of ZIFA, however, is that the zero-inflation model may not be proper for all datasets [4]. Recently, deep learning frameworks, such as Single-cell Variational Inference (scVI) [7] and Sparse Autoencoder for Unsupervised Clustering, Imputation, and Embedding (SAUCIE) [8], utilizes the autoencoder which processes the data through narrower and narrower hidden layers and gradually reduces the dimensionality of the data. It should be noted that scVI and SAUCIE take advantage of parallel and scalable features in deep neural networks [7, 8].

Visualization of high dimensional data is an important problem in scRNA-seq data analysis since it allows us to extract useful information such as distinct cell types. In order to facilitate the process of visualization, dimensionality reduction is normally utilized to reduce the dimension of the data, from tens-of-thousands (that is, the number of genes) to 2 or 3 [2]. T-distributed stochastic neighbor embedding (t-SNE) is a popular method for visualizing scRNA-seq data [9–11], but not recommended as a dimensionality reduction method due to its weaknesses such as curse of intrinsic dimensionality and the infeasibility of handling general dimensionality reduction tasks for a dimensionality higher than three [12]. On the other hand, a recently-developed nonlinear technique called Uniform Manifold Approximation and Projection (UMAP) [13] is claimed to improve visualization of scRNAseq data compared with t-SNE [14].

Generative Adversarial Networks (GANs) [15] are an emerging technique that has attracted much attention in machine learning research because of its massive potential to sample from the true underlying data distribution in a wide variety of applications, such as videos, images, languages, and other fields [16–18]. The GAN framework consists of two components including a generative model *G* and a discriminative model *D* [15]. In practice, these two neural networks, *G* and *D*, are trained simultaneously. The generative model *G* is trained to generate fake samples from the latent variable *z*, while the discriminative model *D* inputs both real and fake samples and distinguishes whether its input is real or not. The discriminative model *D* estimates higher probability if it considers a sample is more likely to be real. In the meantime, *G* is trained to maximize the probability of *D* making a wrong decision. Concurrently, both *G* and *D* play against each other to accomplish their objectives such that the GAN framework creates a min-max adversarial game between *G* and *D*.

Recently, a variant of the GAN framework called an Adversarial AutoEncoder [19] was proposed to be a probabilistic autoencoder that leverages the GAN concept to transform an autoencoder into a GAN-based structure. The architecture of an Adversarial AutoEncoder is composed of two components, a standard autoencoder and a GAN network. The encoder in an Adversarial AutoEncoder is also the generative model of the GAN network. The GAN-based training ensures that the latent space conforms to some prior latent distribution. The Adversarial AutoEncoder models have been applied to identify and generate new compounds for anticancer therapy by using biological and chemical data [20, 21].

The main contributions of this work are as follows: In this work, we propose a novel GAN-based architecture, which we refer to as DR-A (Dimensionality Reduction with Adversarial variational autoencoder), for dimensionality reduction in scRNA-seq analysis. We directly compare the performance of DR-A to dimensionality reduction methods implemented in widely used software, including the PCA, ZIFA, scVI, SAUCIE, t-SNE, and UMAP. Across several scRNA-seq datasets, we demonstrate that our DR-A approach leads to better clustering performance.

## Results

### Overview of DR-A

**Fig. 1:**
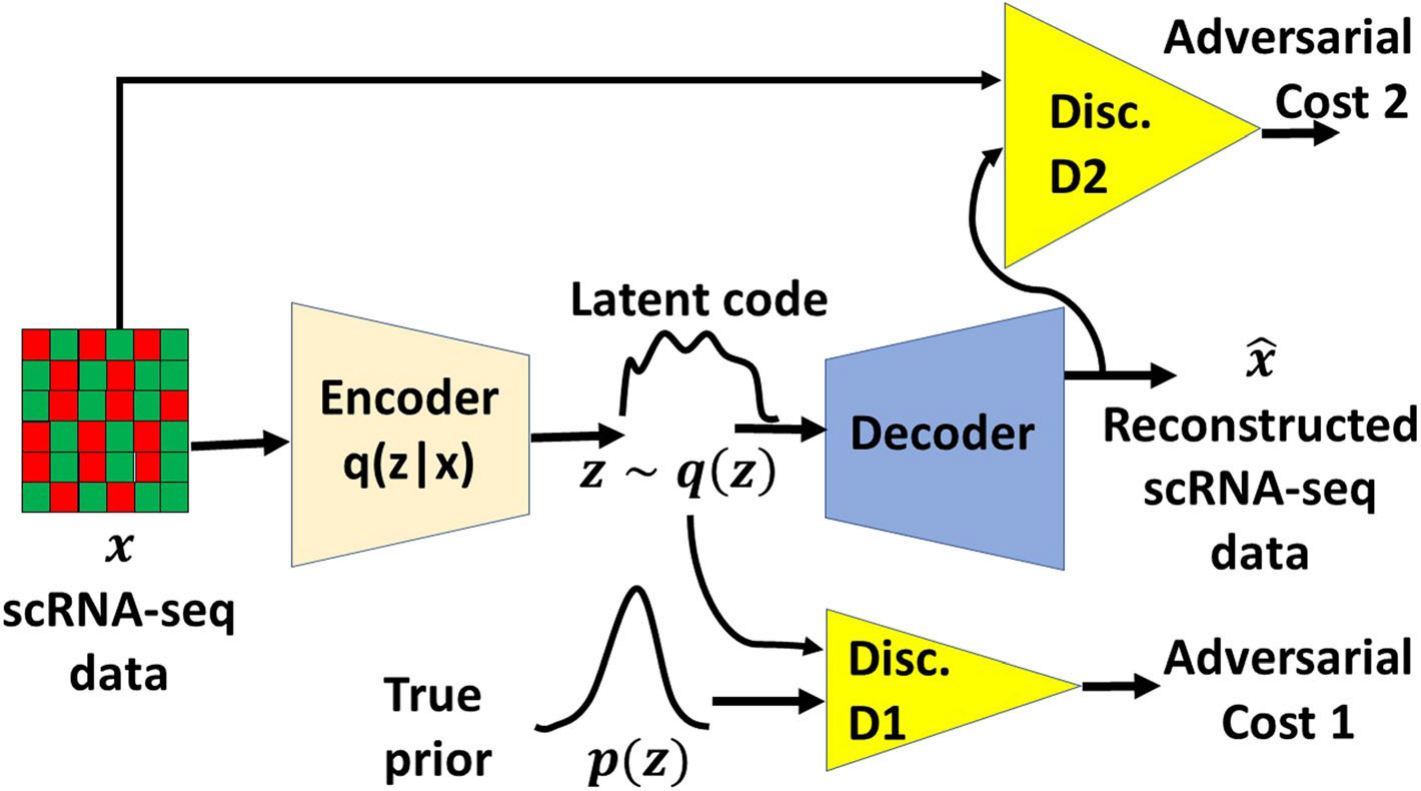
The novel architecture of an *Adversarial Variational AutoEncoder with Dual Matching* (AVAE-DM). An autoencoder (that is, a deep encoder and a deep decoder) reconstructs the scRNA-seq data from a latent code vector *z*. The first discriminator network *D1* is trained to discriminatively predict whether a sample arises from a sampled distribution or from the latent distribution of the autoencoder. The second discriminator *D2* is trained to discriminatively predict whether the scRNA-seq data is real or fake

DR-A represents a deep adversarial variational autoencoder-based framework, which combines the concepts of two deep learning models including Adversarial AutoEncoder [19] and Variational AutoEncoder [22] (see Methods). Figure 1 provides an overview of the model structure in DR-A, which models scRNA-seq data through a zero-inflated negative binomial (ZINB) distribution structure [7, 23] in a GAN framework. DR-A is a novel structure of an *Adversarial Variational AutoEncoder with Dual Matching* (AVAE-DM), where both the generator and discriminator examine the input scRNA-seq data. As shown in Fig. 1, an additional discriminator *D*2 tries to differentiate between real scRNA-seq data and the reconstructed scRNA-seq data from the decoder. While DR-A manages to match the latent space distribution with a selected prior, it concurrently tries to match the distribution of the reconstructed samples with that of the underlying real scRNA-seq data. This approach refers to *dual* distribution *matching*.

In accordance with the Wasserstein distance-based scheme [24], DR-A further integrates the AVAE-DM structure with the Bhattacharyya distance [25]. The Bhattacharyya distance *BD*(*p*, *q*) is an alternative metric to measure the similarity between two probability distributions, *p* and *q* distributions, over the same domain *X*. The Bhattacharyya distance is defined as

$$ \mathrm{BD}\left(p,q\right)=-\ln \left(\sum \limits_{x\in X}\sqrt{p(x)\ast q(x)}\right) $$

Therefore, our new Bhattacharyya distance-based scheme can be formalized as the following minimax objective:

$$ \underset{G}{\min}\underset{D}{\max } BD\left({E}_{x\sim {P}_{data}}\left[D(x)\right],{E}_{z\sim P(z)}\left[D\left(G(z)\right)\right]\right) $$

where *p*_data_ and *p*(*z*) are the data distribution and the model distribution, respectively.

In summary, DR-A has the following five key advantages: (1) DR-A matches the distribution of the reconstructed samples with the underlying real scRNA-seq data. (2) DR-A matches the latent space distribution with a chosen prior. (3) DR-A provides a ZINB distribution, which is a commonly-accepted distributional structure for gene expression. (4) DR-A is more stable for GAN training with the Bhattacharyya distance-based scheme. (5) DR-A accounts for parallel and scalable features in a deep neural network framework (see Methods).

### Real data analysis

To evaluate the performance of our approach for dimension reduction, we compared our DR-A framework with other state-of-the-art methods, including the PCA [3], ZIFA [6], scVI [7], SAUCIE [8], t-SNE [12], and UMAP [13]. The dimensionality reduction was studied in 2 latent dimensions (K = 2), 10 latent dimensions (K = 10), and 20 latent dimensions (K = 20) for these methods.

In these experiments, we employed five datasets (Table 1), including the Zeisel-3 k [1], Macoskco-44 k [10], Zheng-68 k [26], Zheng-73 k [26], and Rosenberg-156 k [27] datasets as described in the Methods section, where the cell types with ground truth are available.

**Table 1:** Summary of scRNA-seq datasets employed in this study. There were 720 highest variance genes selected in each dataset for subsequent experiments

Dataset	Number of cells	Number of cell types	Reference
Zeisel-3 k	3005	7	Zeisel et al. [1]
Macoskco-44 k	44,808	39	Macosko et al. [10]
Zheng-68 k	68,579	10	Zheng et al. [26]
Zheng-73 k	73,233	8	Zheng et al. [26]
Rosenberg-156 k	156,049	73	Rosenberg et al. [27]

We evaluated the effectiveness of these methods with impacts on the clustering performance of the K-means clustering algorithm with the latent dimensions of K = 2, 10, and 20. We assessed the clustering performance using the normalized mutual information (NMI) scores [28]. First, we applied the K-means clustering algorithm using the latent variables from the various algorithms of dimensionality reduction as an input and generated the predicted clustering labels. Then, we utilized NMI scores to measure the cluster purity between the predicted clustering labels and the cell types with ground truth in a given dataset. Based on the NMI scores, we compared our DR-A framework with other algorithms of dimensionality reduction (including the PCA, ZIFA, scVI, SAUCIE, t-SNE, and UMAP methods).

As shown in Table 2, our DR-A framework performed maximally or comparably in all cases. The best NMI scores (with 10 and 20 latent dimensions) for the five datasets were all based on the DR-A method (Table 2(b), K = 10; Table 2(c), K = 20). With 2 latent dimensions, the UMAP method performed marginally better than the DR-A method using the Rosenberg-156 k dataset (Table 2(a), K = 2). In addition, the best NMI scores (with 2 latent dimensions) for the Zheng-73 k, Zheng-68 k, Macosko-44 k, and Zeisel-3 k datasets were all based on the DR-A method (Table 2(a), K = 2).

**Table 2:** Details of experimental results based on NMI scores for various dimension reduction algorithms, including the DR-A, PCA, ZIFA, scVI, SAUCIE, t-SNE, and UMAP methods. We carried out the experiments using the Rosenberg-156 k, Zheng-73 k, Zheng-68 k, Macosko-44 k, and Zeisel-3 k datasets. These dimension reduction algorithms were investigated with (a) 2 latent dimensions (K = 2), (b) 10 latent dimensions (K = 10), and (c) 20 latent dimensions (K = 20)

Algorithm	Rosenberg-156 k	Zheng-73 k	Zheng-68 k	Macosko-44 k	Zeisel-3 k
(a) K = 2					
DR-A	0.5573	**0.8457**	**0.5931**	**0.4936**	**0.7263**
PCA	0.2523	0.3396	0.2538	0.2984	0.4721
ZIFA	0.3049	0.3794	0.2810	0.3120	0.4250
scVI	0.5199	0.8261	0.5417	0.4599	0.7006
SAUCIE	0.4046	0.4304	0.2749	0.2707	0.4622
t-SNE	0.4343	0.6562	0.4081	0.4091	0.7103
UMAP	**0.5591**	0.6507	0.4377	0.4184	0.7214
(b) K = 10					
DR-A	**0.5850**	**0.8503**	**0.5756**	**0.5156**	**0.7893**
PCA	0.3276	0.5612	0.3877	0.4243	0.5559
ZIFA	0.5074	0.8354	0.5152	0.4785	0.7807
scVI	0.5821	0.8060	0.5571	0.5155	0.7606
SAUCIE	0.4773	0.4209	0.3147	0.2874	0.5110
t-SNE	N/A	N/A	N/A	N/A	N/A
UMAP	0.5735	0.6911	0.4393	0.4129	0.7413
(c) K = 20					
DR-A	**0.5842**	**0.8002**	**0.5888**	**0.5176**	**0.7639**
PCA	0.3761	0.5623	0.3874	0.4306	0.5561
ZIFA	N/A	N/A	N/A	N/A	0.7114
scVI	0.5831	0.7976	0.5691	0.5105	0.7419
SAUCIE	0.4740	0.4254	0.2952	0.2775	0.4808
t-SNE	N/A	N/A	N/A	N/A	N/A
UMAP	0.5656	0.6906	0.4413	0.4177	0.7419

N/A denotes that we could not run the given algorithm

Furthermore, we compared our DR-A framework with other variants of the GAN framework, including the AVAE-DM structure with the Wasserstein distance and AVAE structure. Our DR-A framework adopts the AVAE-DM structure with Bhattacharyya distance. The DR-A method improved the performance compared to the AVAE-DM with the Wasserstein distance and AVAE methods (Additional file 1: Table S1), indicating the advantage of the Bhattacharyya distance and dual matching architecture. In addition, the experimental results of the DR-A method with various batch sizes were shown in Additional file 1: Table S2.

Our analysis indicated that our DR-A framework is well-suited for large-scale scRNA-seq datasets. The hyperparameters for various datasets of DR-A were shown in Table 3.

**Table 3:** Details of hyperparameters for DR-A based on the experimental results in Table 2. We carried out the experiments using the Rosenberg-156 k, Zheng-73 k, Zheng-68 k, Macosko-44 k, and Zeisel-3 k datasets. The DR-A algorithm was investigated with (a) 2 latent dimensions (K = 2), (b) 10 latent dimensions (K = 10), and (c) 20 latent dimensions (K = 20). *G* denotes a generative model and *D* denotes a discriminative model

Dataset	Batch size	Hidden layer	Hidden unit	Learning rate
(a) K = 2				
Rosenberg-156 k	128	4	*G*: 1024/512/512/256 *D*: 32/16/16/8	7 × 10^−5^
Zheng-73 k	128	3	*G*: 512/512/512 *D*: 32/32/32	6 × 10^−5^
Zheng-68 k	128	4	*G*: 256/256/256/256 *D*: 32/32/16/16	0.0001
Macosko-44 k	128	3	*G*: 256/128/64 *D*: 64/64/64	0.0001
Zeisel-3 k	128	4	*G*: 512/512/512/512 *D*: 32/32/32/32	8 × 10^−4^
(b) K = 10				
Rosenberg-156 k	128	4	*G*: 512/256/128/64 *D*: 256/128/64/32	6 × 10^−5^
Zheng-73 k	128	4	*G*: 1024/512/512/256 *D*: 32/32/32/32	2 × 10^−5^
Zheng-68 k	128	4	*G*: 256/256/256/256 *D*: 32/32/16/16	7 × 10^−5^
Macosko-44 k	128	4	*G*: 512/256/256/128 *D*: 256/128/128/64	7 × 10^−5^
Zeisel-3 k	128	1	*G*: 512 *D*: 512	7 × 10^−4^
(c) K = 20				
Rosenberg-156 k	128	4	*G*: 1024/1024/1024/1024 *D*: 64/64/64/64	6 × 10^−5^
Zheng-73 k	128	4	*G*: 1024/512/512/256 *D*: 64/32/32/16	1 × 10^−5^
Zheng-68 k	128	1	*G*: 256 *D*: 256	2 × 10^−5^
Macosko-44 k	128	1	*G*: 256 *D*: 256	7 × 10^−5^
Zeisel-3 k	128	1	*G*: 512 *D*: 512	7 × 10^−4^

### Data visualization

**Fig. 2:**
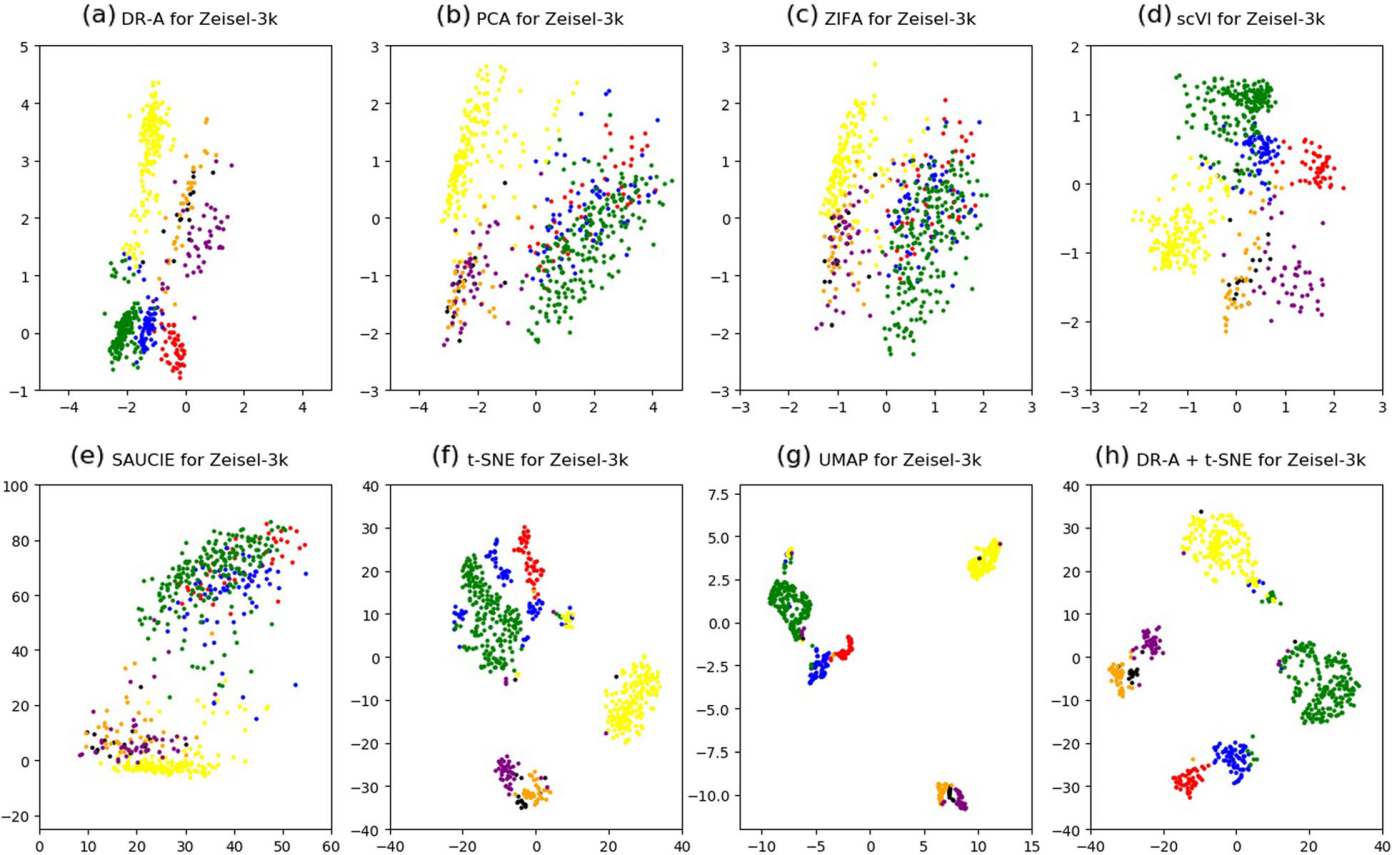
2-D visualization for the Zeisel-3 k dataset. The Zeisel-3 k dataset was reduced to 2-D by using (**a**) DR-A, (**b**) PCA, (**c**) ZIFA, (**d**) scVI, (**e**) SAUCIE, (**f**) t-SNE, (**g**) UMAP, and (**h**) DR-A combined with t-SNE methods. Each point in the 2-D plot represents a cell in the testing set of the Zeisel dataset, which have 7 distinct cell types. There was an 80% training and 20% testing split from the original dataset in these experiments

**Fig. 3:**
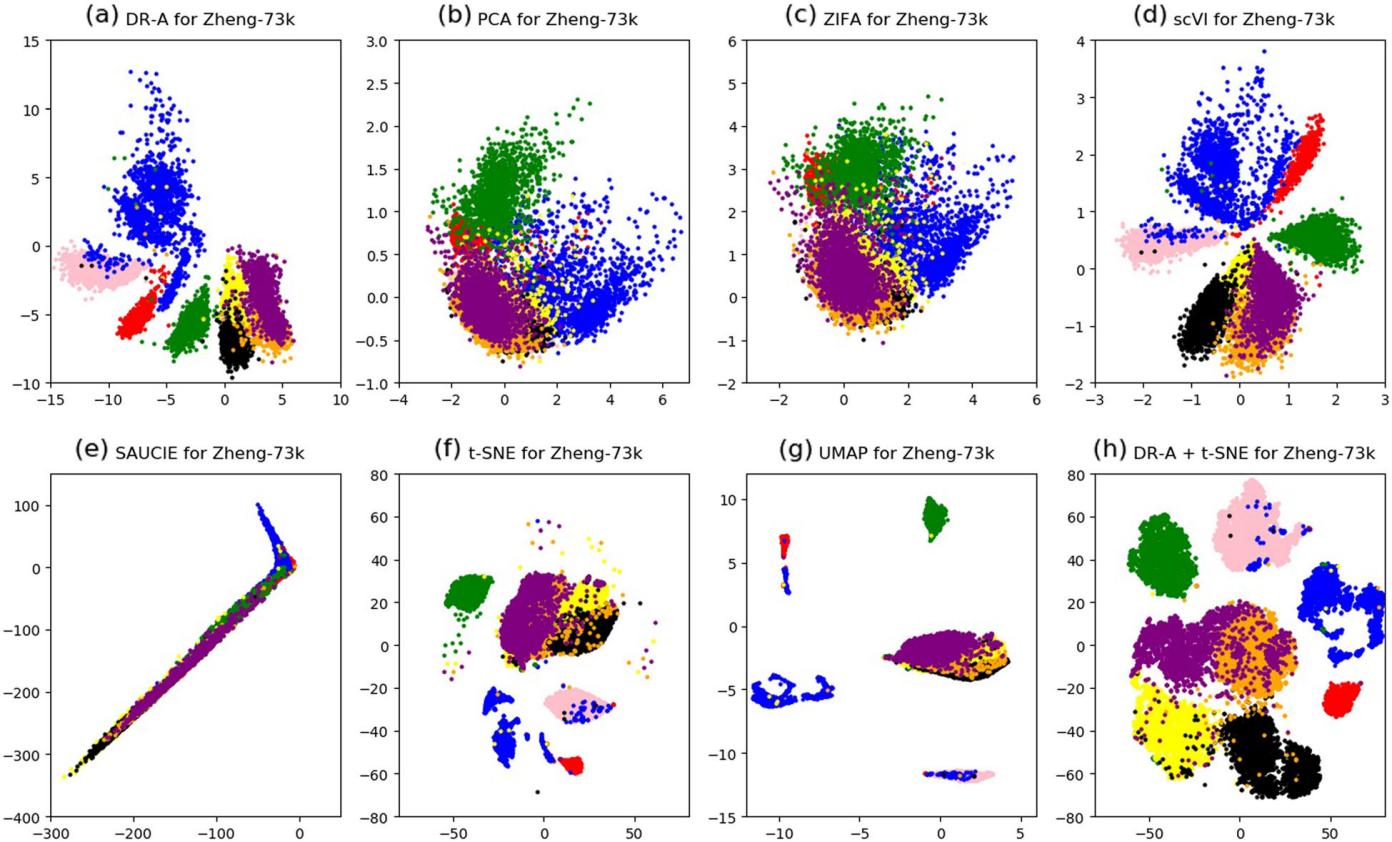
2-D visualization for the Zheng-73 k dataset. The Zheng-73 k dataset was reduced to 2-D by using (**a**) DR-A, (**b**) PCA, (**c**) ZIFA, (**d**) scVI, (**e**) SAUCIE, (**f**) t-SNE, (**g**) UMAP, and (**h**) DR-A combined with t-SNE methods. Each point in the 2-D plot represents a cell in the testing set of the Zheng-73 k dataset, which have 8 distinct cell types. There was an 80% training and 20% testing split from the original dataset in these experiments

Moreover, we performed two-dimensional (2-D) visualization of the clustering results for the DR-A, PCA, ZIFA, scVI, SAUCIE, t-SNE, and UMAP methods using the Zeisel-3 k (Fig. 2), Zheng-73 k (Fig. 3), Macoskco-44 k (Additional file 1: Figure S1), Zheng-68 k (Additional file 1: Figure S2), and Rosenberg-156 k (Additional file 1: Figure S3) datasets, respectively. We also carried out the two-step approach of combining DR-A with t-SNE (see Methods). We illustrated the 2-D plots on the Macoskco-44 k (Additional file 1: Figure S1) and Rosenberg-156 k datasets (Additional file 1: Figure S3) only by using the top ten cell types in terms of the number of cells. Due to the large number of distinct cell types for the Macoskco-44 k and Rosenberg-156 k datasets (39 and 73, respectively), it may not be obvious to distinguish in 2-D visualization by using all cell types.

## Discussion

In this work, we specifically addressed the problem of the identification of distinct cellular subtypes in terms of dimensionality reduction in scRNA-seq data. We developed a conceptually different class of the GAN framework, DR-A, which is an AVAE-DM-based method for robust estimation of cell types and is applicable to large-scale scRNA-seq datasets. We further demonstrated the utility of DR-A in an application to five real scRNA-seq datasets assuming 2, 10, and 20 latent dimensions. We also compared the performance of DR-A to state-of-the-art methods and intriguingly showed the improvement offered by DR-A over widely used approaches, including PCA, ZIFA, scVI, SAUCIE, t-SNE, and UMAP.

Furthermore, our experiments demonstrated that our DR-A framework, which is based on the AVAE-DM model with the Bhattacharyya distance, is a promising novel approach. All in all, our DR-A method had a better performance than state-of-the-art methods for all five datasets, indicating that DR-A is scalable for large-scale scRNA-seq datasets.

Although the t-SNE method is a wide-used approach for data visualization of scRNA-seq data, it has been suggested that t-SNE may not be feasible for dimensionality reduction [12]. In line with this finding in the previous study, the clustering performances of t-SNE in some datasets were worse than those of other algorithms such as scVI and DR-A in this study (Table 2). To overcome this weakness, some studies [10] utilized a technique of using t-SNE for data visualization after performing other dimensionality reduction methods. In accordance with this technique, we adapted the two-step approach of using DR-A with t-SNE. Interestingly, we found that the two-step approach combines the advantages of both DR-A and t-SNE methods and had an improved result that cells from relevant cell types appeared to be adjacent to each other, for example, as shown in Fig. 2 (a), (f), and (h) for the Zeisel-3 k dataset. Likewise, the improvement for data visualization is presented for other four datasets (Fig. 3, Additional file 1: Figure S1, Additional file 1: Figure S2, and Additional file 1: Figure S3). Therefore, our results demonstrate that DR-A is an effective 2-D visualization tool for scRNA-seq data.

## Conclusions

In summary, we developed DR-A, a novel AVAE-DM-based framework, for scRNA-seq data analysis and applications in dimension reduction and clustering. Compared systematically with other state-of-the-art methods, DR-A achieves higher cluster purity for clustering tasks and is generally suitable for different scale and diversity of scRNA-seq datasets. We anticipate that scalable tools such as DR-A will be a complementary approach to existing methods and will be in great demand due to an ever-increased need for handling large-scale scRNA-seq data. In future work, we will verify if DR-A could also be beneficial for other forms of downstream analysis, such as lineage estimation.

## Methods

### Generative adversarial networks

The idea of GANs is to train two neural networks (the generator *G* and the discriminator *D*) concurrently to establish a min-max adversarial game between them. The generator *G*(*z*) gradually learns to transform samples *z* from a prior distribution *p*(*z*) into the data space, while the discriminator *D*(x) is trained to distinguish a point x in the data space between the data points sampled from the actual data distribution (that is, true samples) and the data points produced by the generator (that is, fake samples). It is assumed that *G*(*z*) is trained to fully confuse the discriminator with its generated samples by using the gradient of *D*(x) with respect to x to modify its parameters. This scheme can be formalized as the following type of minimax objective [15]:

$$ \underset{G}{\min}\underset{D}{\max }{E}_{x\sim {P}_{data}}\left[\log D(x)\right]+{E}_{z\sim P(z)}\left[\log \left(1-D\left(G(z)\right)\right)\right] $$

where *p*_data_ is the data distribution and *p*(*z*) is the model distribution.

The generator *G* and the discriminator *D* can be both modeled as fully connected neural networks and then are trained by backpropagation using a suitable optimizer. In our experiments, we used adaptive moment estimation (Adam) [29], which is an extension to stochastic gradient descent.

### Adversarial AutoEncoder

A variant of GAN models called an Adversarial AutoEncoder [19] is a probabilistic autoencoder that transforms an autoencoder into a generative model by using the GAN framework. The structure of an Adversarial AutoEncoder is composed of two components, a standard autoencoder and an adversarial network. The encoder is also the generator of the adversarial network. The idea of the Adversarial AutoEncoder is that both the adversarial network and the autoencoder are trained simultaneously to perform inference. While the encoder (that is, the generator) is trained to fool the discriminator to believe that the latent vector is generated from the true prior distribution, the discriminator is trained to distinguish between the sampled vector and the latent vector of the encoder at the same time. The adversarial training ensures that the latent space matches with some prior latent distribution.

### Variational AutoEncoder

A variant of autoencoder models called Variational Autoencoder [22] is a generative model, which estimates the probability density function of the training data. An input *x* is run through an encoder, which generates parameters of a distribution *Q*(*z* | *x*). Then, a latent vector *z* is sampled from *Q*(*z* | *x*). Finally, the decoder decodes *z* into an output, which should be similar to the input. This scheme can be trained by maximizing the following objective with gradient-based methods:

$$ {E}_{z\sim Q\left(z|x\right)}\ \log {p}_{model}\left(x\ |\ z\right)-{D}_{KL}\left(Q\left(z\ \right|x\right)\left\Vert {p}_{model}(z)\right) $$

where *D*_*KL*_ is the Kullback–Leibler divergence, and *p*_*model*_(*x* | *z*) is viewed as the decoder.

### Adversarial Variational AutoEncoder

**Fig. 4:**
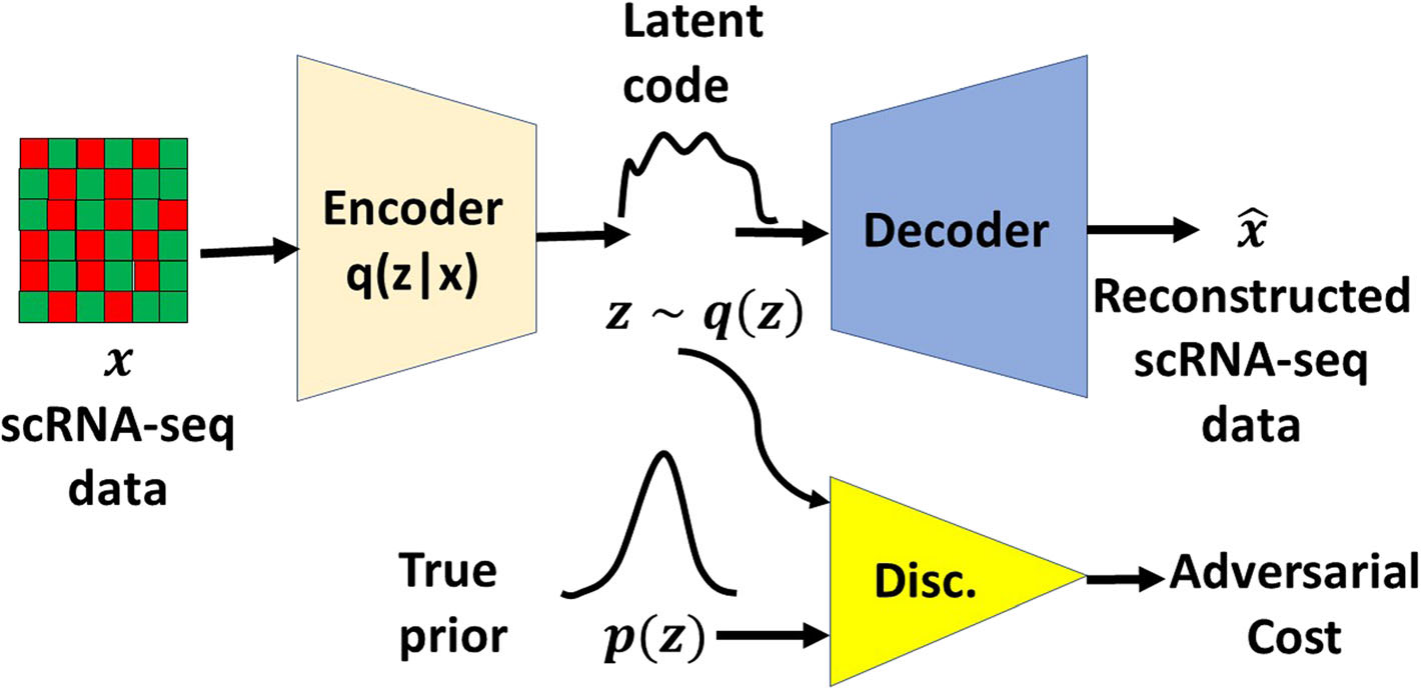
The overall architecture of an Adversarial Variational AutoEncoder (AVAE) framework. An autoencoder (that is, a deep encoder and a deep decoder) reconstructs the scRNA-seq data from a latent code vector *z*. A discriminator network is trained to discriminatively predict whether a sample arises from a prior distribution or from the latent code distribution of the autoencoder

Figure 4 shows the structure of an Adversarial Variational AutoEncoder (AVAE), which adopts the structures of Adversarial Autoencoder [19] and Variational Autoencoder [22]. Let *x* be the input of the scRNA-seq expression level (*M* cells x *N* genes) and *z* be the latent code vector of an autoencoder, which consists of a deep encoder and a deep decoder. Let *p*(*z*) be the prior distribution imposed on the latent code vector, *q*(*z*|*x*) be an encoding distribution and *p*(*x*|*z*) be the decoding distribution. The deep encoder provides the mean and covariance of Gaussian for the variational distribution *q*(*z*|*x*) [22]. The autoencoder gradually learns to reconstruct the input *x* of the scRNA-seq data to be as realistic as possible by minimizing the reconstruction error. Note that the encoder of the AVAE is also the generator of the GAN framework. The encoder is trained to fool the discriminator of the GAN framework such that the latent code vector *q*(*z*) stems from the true prior distribution *p*(*z*). Meanwhile, the discriminator is trained to distinguish between the sampled vector of *p*(*z*) and the latent code vector *q*(*z*) of the encoder (that is, the generator) at the same time. Thus, the GAN framework guides *q*(*z*) to match *p*(*z*). Eventually, AVAE is able to learn an unsupervised representation of the probability distribution of the scRNA-seq data. In our work, we used the normal Gaussian distribution *N*(0, **I**) for the prior distribution *p*(*z*). In addition, the generator was updated twice for each discriminator update in this work. Note that in the training phase, labels for cell types are not provided and the entire framework is unsupervised.

### Adversarial Variational AutoEncoder with dual matching (AVAE-DM)

In this paper, we explore AVAEs in a different structure by altering the network architecture of an AVAE (Fig. 4). Figure 1 shows the novel structure of an *Adversarial Variational AutoEncoder with Dual Matching* (AVAE-DM) employed in this work. Unlike a conventional AVAE, both the generator and discriminator observe the input scRNA-seq data in an AVAE-DM. In additional to the original AVAE structure (Fig. 4), we add another discriminator *D*2 that attempts to distinguish between real scRNA-seq data and the decoder’s output (that is, the reconstructed scRNA-seq data). As in the original AVAE structure, the goal of this AVAE-DM architecture remains the same in the unsupervised setting (that is, labels for cell types are not provided during training). This architecture ensures that the distribution of the reconstructed samples match that of the underlying real scRNA-seq. At the same time, the latent space distribution is matched with a chosen prior, leading to *dual* distribution *matching*.

Since the Wasserstein distance have been shown to be more stable for GAN training, the AVAE-DM can be combined with the Wasserstein distance [30]. The AVAE-DM can also be explored with the Wasserstein distance with gradient penalty (GP) [24]. Wasserstein distance *W*(*p*, *q*), also known as the earth mover’s distance, is informally defined as the minimum cost of transiting mass between the probability distribution *p* and the probability distribution *q*. The Wasserstein distance-based scheme can be formalized as the following minimax objective [24]:

$$ \underset{G}{\min}\underset{D}{\max }{E}_{x\sim {P}_{data}}\left[D(x)\right]-{E}_{z\sim P(z)}\left[D\left(G(z)\right)\right] $$

Furthermore, we proposed to integrate the AVAE-DM with the Bhattacharyya distance [25], which is yet another metric to measure the similarity of two probability distributions. The Bhattacharyya distance *BD*(*p*, *q*) between *p* and *q* distributions over the same domain *X* is defined as

$$ \mathrm{BD}\left(p,q\right)=-\ln \left(\sum \limits_{x\in X}\sqrt{p(x)\ast q(x)}\right) $$

Then, our new objective is

$$ \underset{G}{\min}\underset{D}{\max } BD\left({E}_{x\sim {P}_{data}}\left[D(x)\right],{E}_{z\sim P(z)}\left[D\left(G\left(\mathrm{z}\right)\right)\right]\right) $$

where *p*_data_ and *p*(*z*) are once again the data distribution and the model distribution, respectively.

Our DR-A approach mainly encompasses the AVAE-DM-based algorithm with Bhattacharyya distance. In DR-A, we employed ZINB conditional likelihood for *p*(*x*|*z*) to reconstruct the decoder’s output for the scRNA-seq data [7, 23]. To accordingly handle dropout events (that is, zero expression measurements), DR-A models the scRNA-seq expression level *x* following a ZINB distribution, which appears to provide a good fit for the scRNA-seq data [7, 23].

In this study, the encoder, decoder, and discriminator are designed from 1, 2, 3, or 4 layers of a fully connected neural network with 8, 16, 32, 64, 128, 256, 512, or 1024 nodes each. The best hyper-parameter set from numerous possibilities was chosen from a grid search that maximized clustering performance in the testing data sets. Dropout regularization was used for all neural networks. The activation functions between two hidden layers are all leaky rectified linear (Leaky ReLu) activation functions. Deep learning models have high variance and never give the same answer when running multiple times. In order to achieve reproducible results, we used the Python and TensorFlow commands such as np.random.seed(0) and tf.set_random_seed(0) to obtain a single number.

### Benchmarking

For the benchmarking task, we employed several state-of-the-art methods as described below. We employed the ZIFA method [6] with the block algorithm (that is, function block) using default parameters, which is implemented in the ZIFA python package (Version 0.1) and is available at https://github.com/epierson9/ZIFA. The outcome of ZIFA is an N x K matrix corresponding to a low-dimensional projection in the latent space with the number of samples N and the number of latent dimensions K, where we chose K = 2, 10, and 20.

Furthermore, we used the PCA method [3] from Scikit-learn, a machine learning library, using default parameters and log-data. We also employed the t-SNE method [12] from Scikit-learn, a machine learning library, using default parameters (for example, perplexity parameter of 30). In addition, we utilized the UMAP method [13], a manifold learning technique, using default parameters and log-data. The embedding layer was 2 10, and 20 latent dimensions.

Moreover, we utilized scVI [7], which is based on the variational autoencoder [22] and conditional distributions with a ZINB form [31]. Based on the implications described in scVI [7], we used one layer with 128 nodes in the encoder and one layer with 128 nodes in the decoder. We also used two layers with 128 nodes in the encoder and two layers with 128 nodes in the decoder. The embedding layer was 2, 10, and 20 latent dimensions. The ADAM optimizer was used with learning rate 0.001. The hyper-parameters were selected through best clustering performance in the testing data.

We also employed SAUCIE [8], which is based on the autoencoder [32]. SAUCIE consists of an encoder, an embedding layer, and then a decoder. Based on the indications reported in SAUCIE [8], we used three layers with 512, 256, and 128 nodes in the encoder and symmetrically three layers with 128, 256, and 512 nodes in the decoder. We also used three layers with 256, 128, and 64 nodes in the encoder and symmetrically three layers with 64, 128, and 256 nodes in the decoder. The embedding layer was 2 10, and 20 latent dimensions. The ADAM optimizer was used with learning rate 0.001. The hyper-parameters were chosen via best clustering performance in the testing data sets.

### Datasets

Table 1 shows the list of the five scRNA-seq datasets used in this study. All datasets were pre-processed to obtain 720 highest variance genes across the cells [33]. It is assumed that genes with highest variance relative to their mean expression are as a result of biological effects instead of technical noise [4]. The transformation used in the counts matrix data *C* was log_2_ (1 + *C*).

As shown in Table 1, the Zeisel-3 k dataset [1] consists of 3005 cells in the somatosensory cortex and hippocampal region from the mouse brain. The Zeisel-3 k dataset has the ground truth labels of 7 distinct cell types such as pyramidal cells, oligodendrocytes, mural cells, interneurons, astrocytes, ependymal cells, and endothelial cells in the brain.

Moreover, the Macoskco-44 k dataset [10] is comprised of cells in the mouse retina region and chiefly consists of retinal cell types such as amacrine cells, bipolar cells, horizontal cells, photoreceptor cells, and retinal ganglion cells. In addition, the Zheng-68 k dataset [26] contains fresh peripheral blood mononuclear cells in a healthy human and principally involves major cell types of peripheral blood mononuclear cells such as T cells, NK cells, B cells, and myeloid cells. Furthermore, the Zheng-73 k dataset [26] consists of fluorescence-activated cell sorting cells in a healthy human and primarily incorporates T cells, NK cells, and B cells. Finally, the Rosenberg-156 k dataset [27] consists of cells from mouse brains and spinal cords and mainly contains neuronal cell types such as cerebellar granule cells, mitral cells, and tufted cells.

### Performance evaluation

In order to evaluate the quality of low-dimensional representation from dimension reduction, we applied the K-means clustering algorithm to the low-dimensional representations of the dimension reduction methods (including the DR-A, PCA, scVI, SAUCIE, ZIFA, t-SNE, and UMAP methods as described previously) and compared the clustering results to the cell types with ground truth labels, where we set the number of clusters to the number of cell types. Then, we employed NMI scores [28] to assess the performance. Assume that *X* is the predicted clustering results and *Y* is the cell types with ground truth labels, NMI is calculated as follows:

$$ \mathrm{NMI}=\frac{MI\left(X;Y\right)}{\sqrt{H(X)H(Y)}} $$

where *MI* is the mutual entropy between *X* and *Y*, and *H* is the Shannon entropy.

### Data visualization

After we performed the dimensionality reduction task using our DR-A framework, we leveraged the low-dimensional view of the data for visualization. The objective of the visualization task is to identify cell types in an un-labelled dataset and then display them in 2-D space. Note that all our datasets had a training set and a testing set with an 80% training and 20% testing split from the original dataset. First, we trained our DR-A model to perform the clustering task in 2 latent dimensions (K = 2) using the training set. Next, we obtained a two-dimensional embedding (K = 2) of the scRNA-seq data by projecting the testing set with the trained DR-A model. This latent (K = 2) estimated by our DR-A model represents two dimensional coordinates for each input data point, which was then utilized to perform a 2-D plot. Similarly, we implemented 2-D plots for the PCA, ZIFA, scVI, SAUCIE, t-SNE, and UMAP methods after performing the clustering task in 2 latent dimensions (K = 2), respectively.

In addition, we performed data visualization by a two-step approach, which combines our DR-A method with the t-SNE algorithm. In the first step, we performed the clustering task in 10 latent dimensions (K = 10) using our DR-A model. In the second step, we used the latent (K = 10) estimated in the first step as input to the t-SNE algorithm and generated a two-dimensional embedding (K = 2) of the scRNA-seq data. This latent (K = 2) estimated by the t-SNE algorithm represents two dimensional coordinates for each input data point, which was then utilized to perform a 2-D plot.

## Supplementary information

**Additional file 1.** Supplementary tables and figures. This PDF file contains additional tables and figures related to this manuscript.

## Data Availability

The datasets and source code that support the findings of this study are available in https://github.com/eugenelin1/DRA.

## Abbreviations

2-D: Two-dimensional
AVAE-DM: Adversarial Variational AutoEncoder with Dual Matching
DR-A: Dimensionality Reduction with Adversarial variational autoencoder
GANs: Generative Adversarial Networks
NMI: Normalized mutual information
PCA: Principal component analysis
SAUCIE: Sparse Autoencoder for Unsupervised Clustering, Imputation, and Embedding
scRNA-seq: single-cell RNA sequencing
scVI: Single-cell Variational Inference
t-SNE: t-distributed stochastic neighbor embedding
UMAP: Uniform Manifold Approximation and Projection
ZIFA: Zero-Inflated Factor Analysis
ZINB: Zero-inflated negative binomial
