# Supplementary material for: A deep adversarial variational autoencoder model for dimensionality reduction in single-cell RNA sequencing analysis
Source: BMC Bioinformatics. 2020 Feb 21;21:64. doi: 10.1186/s12859-020-3401-5 (PMC7035735; doi:10.1186/s12859-020-3401-5)
Supplement: Supplementary file 1 — Additional file 1. Supplementary tables and figures. This PDF file contains additional tables and figures related to this manuscript. [file 12859_2020_3401_MOESM1_ESM.pdf]

This file contains Supplementary Tables and Supplementary Figures

for

A deep adversarial variational autoencoder model for dimensionality reduction in single-cell RNA sequencing analysis

Eugene Lin[1,2,3], Sudipto Mukherjee[1], Sreeram Kannan[1]*

[1] Department of Electrical & Computer Engineering, University of Washington, Seattle, WA 98195, USA

[2] Department of Biostatistics, University of Washington, Seattle, WA 98195, USA

[3] Graduate Institute of Biomedical Sciences, China Medical University, Taichung, Taiwan

* *Corresponding author*:

Dr. Sreeram Kannan, Department of Electrical & Computer Engineering, University of Washington, Seattle, WA 98195, USA

E-mail: ksreeram@uw.edu

Table S1. Details of experimental results based on NMI scores for various dimension reduction algorithms, including the DR-A, AVAE-DM with the Wasserstein distance, and AVAE methods. We carried out the experiments using the Rosenberg-156k, Zheng-73k, Zheng-68k, Macosko-44k, and Zeisel-3k datasets. These dimension reduction algorithms were investigated with 2 latent dimensions (K = 2).

Algorithm	Rosenberg-156k	Zheng-73k	Zheng-68k	Macosko-44k	Zeisel-3k
DR-A	0.5573	0.8457	0.5931	0.4936	0.7263
AVAE-DM with the Wasserstein distance	0.1492	0.1586	0.4520	0.4068	0.4200
AVAE	0.4822	0.7622	0.5712	0.4641	0.6211

The DR-A approach is mainly based on the AVAE-DM algorithm with Bhattacharyya distance.

AVAE = Adversarial Variational AutoEncoder; AVAE-DM: Adversarial Variational AutoEncoder with Dual Matching.

Table S2. Details of experimental results based on NMI scores for various batch sizes using the DR-A method. We carried out the experiments using the Rosenberg-156k, Zheng-73k, Zheng-68k, Macosko-44k, and Zeisel-3k datasets. These batch sizes were investigated with 2 latent dimensions (K = 2).

Batch size	Rosenberg-156k	Zheng-73k	Zheng-68k	Macosko-44k	Zeisel-3k
64	0.5107	0.7513	0.5844	0.4694	0.6564
128	0.5573	0.8457	0.5931	0.4936	0.7263
256	0.5119	0.5932	0.4517	0.4781	0.5389
512	0.4353	0.6381	0.4053	0.4103	0.5162
1024	0.4538	0.5782	0.3719	0.3640	0.5331

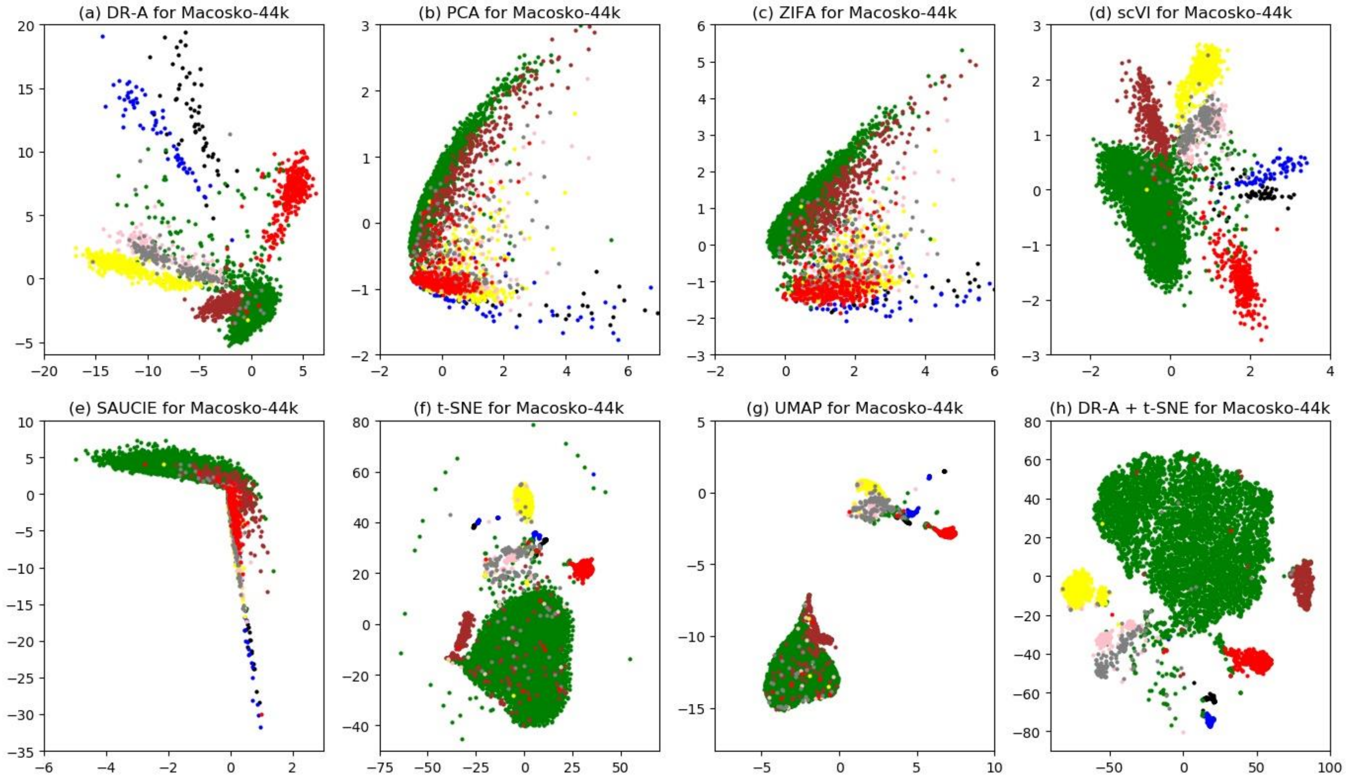

Figure S1. 2-D visualization for the Macoskco-44k dataset. The Macoskco-44k dataset was reduced to 2-D by using (a) DR-A, (b) PCA, (c) ZIFA, (d) scVI, (e) SAUCIE, (f) t-SNE, (g) UMAP, and (h) DR-A combined with t-SNE methods. Each point in the 2-D plot represents a cell in the testing set of the Macoskco-44k dataset, which have 39 distinct cell types. There was an 80% training

and 20% testing split from the original dataset in these experiments. Because there are many distinct cell types for the Macoskco-44k dataset, this 2-D plot only shows the top ten cell types based on the number of cells.

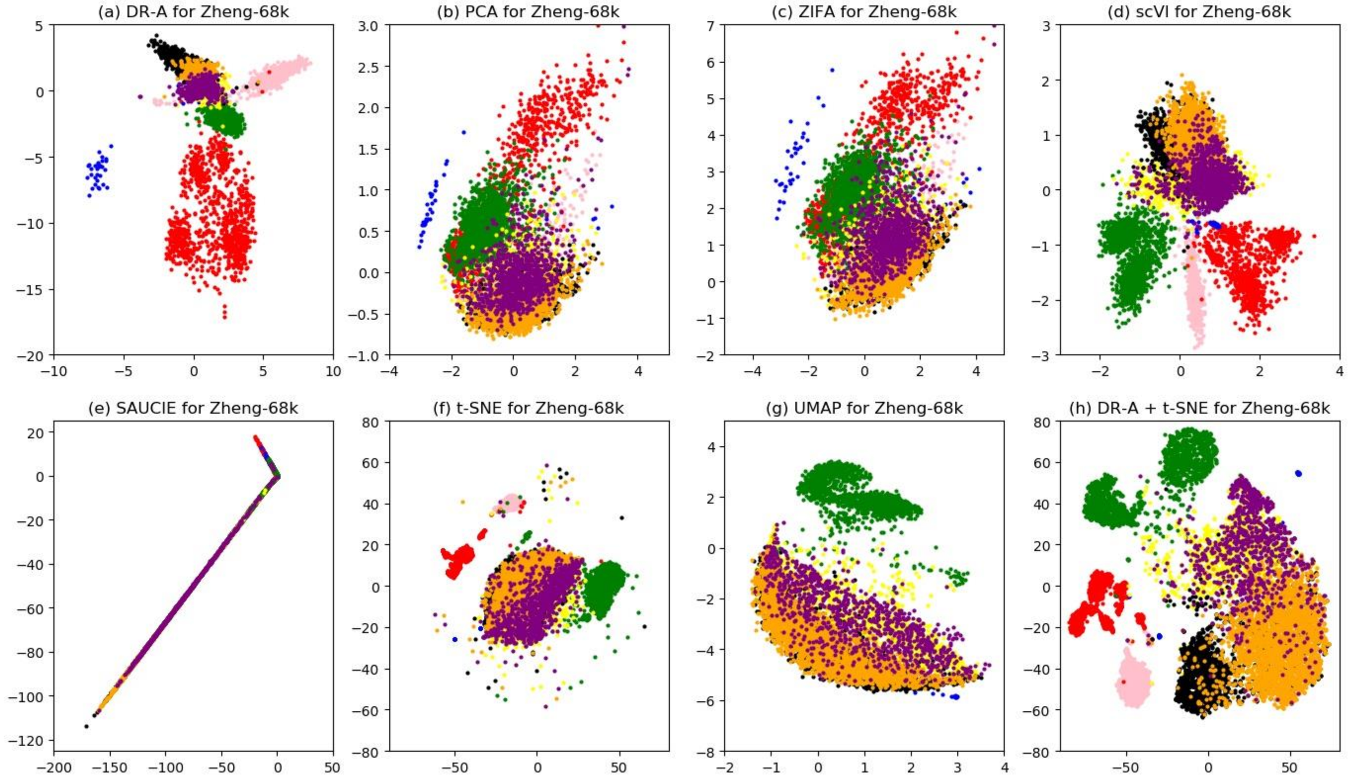

Figure S2. 2-D visualization for the Zheng-68k dataset. The Zheng-68k dataset was reduced to 2-D by using (a) DR-A, (b) PCA, (c) ZIFA, (d) scVI, (e) SAUCIE, (f) t-SNE, (g) UMAP, and (h) DR-A combined with t-SNE methods. Each point in the 2-D plot represents a cell in the testing set of the Zheng-68k dataset, which have 10 distinct cell types. There was an 80% training and 20%

testing split from the original dataset in these experiments.

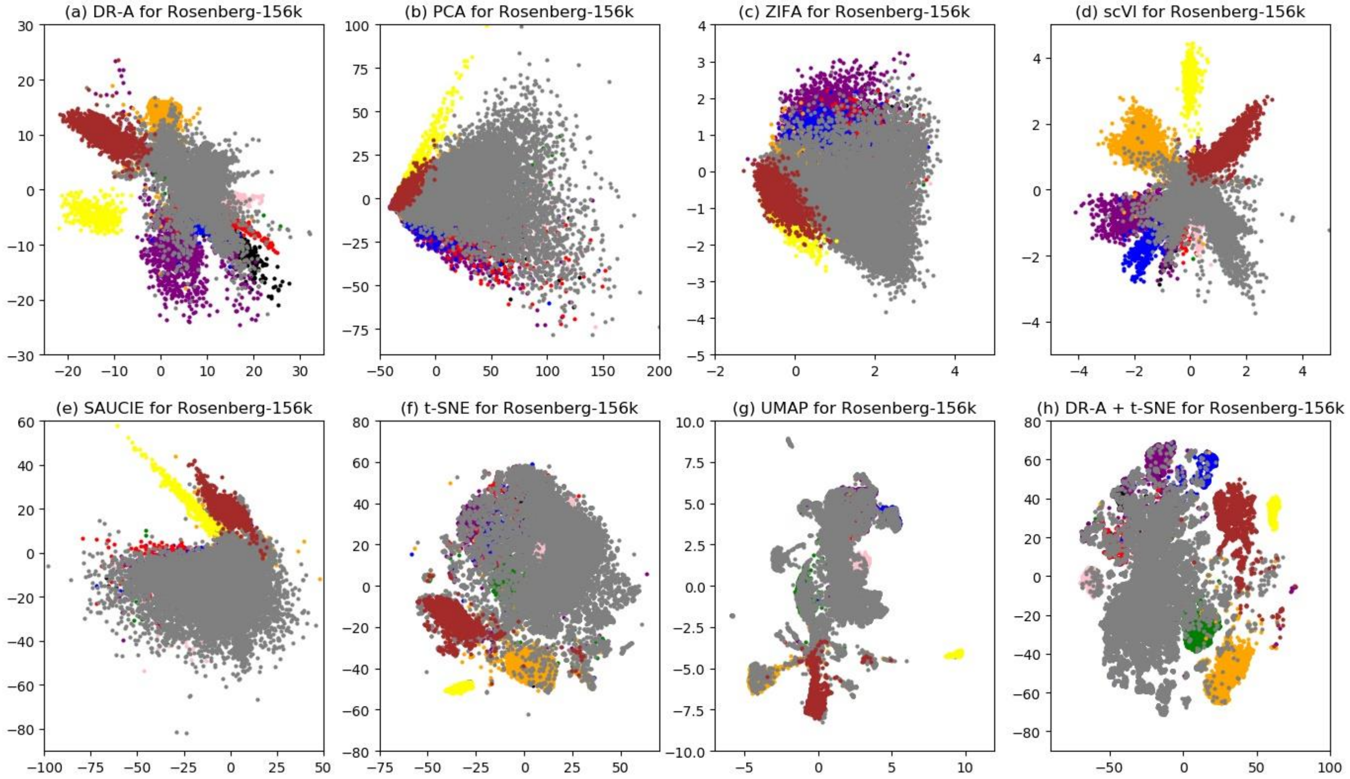

Figure S3. 2-D visualization for the Rosenberg-156k. The Rosenberg-156k dataset was reduced to 2-D by using (a) DR-A, (b) PCA, (c) ZIFA, (d) scVI, (e) SAUCIE, (f) t-SNE, (g) UMAP, and (h) combined with t-SNE methods. Each point in the 2-D plot represents a cell in the testing set of the Rosenberg-156k dataset, which have 73 distinct cell types. There was an 80% training and

20% testing split from the original dataset in these experiments. Because there are many distinct cell types for the Rosenberg-156k dataset, this 2-D plot only shows the top ten cell types based on the number of cells.
